# Supplementary material for: Motor Control Evaluation as a Significant Component in Upper Limb Function Assessment in Female Breast Cancer Patients after Mastectomy
Source: Healthcare (Basel). 2021 Jul 31;9(8):973. doi: 10.3390/healthcare9080973 (PMC8391901; doi:10.3390/healthcare9080973)
Supplement: Supplementary file 1 [file healthcare-09-00973-s001.zip › Table S1 The history examination card.pdf]

1.Examination’s date:

2. Basic information:

Name:

Surname:

Age:

Weight:

Sex:

Handiness:

Did you suffer from pain / injures / fractures / luxation of shoulder complex int the past ?

3.Information about your treatment

Side of surgery:

Type of surgery:

Time from surgery:

Use of radiation therapy:

Yes / No

Before surgery / after surgery / both

Use of chemotherapy:

Yes / No

Shoulder complex pain on the site of surgery:

Yes / No

Did you feel pain in shoulder complex before the surgery (on the site of surgery)?

Yes / No

Did you have lymphedema after surgery?

Yes / No
