# Supplementary figures and images for: Motor Control Evaluation as a Significant Component in Upper Limb Function Assessment in Female Breast Cancer Patients after Mastectomy
Source: Healthcare (Basel). 2021 Jul 31;9(8):973. doi: 10.3390/healthcare9080973 (PMC8391901; doi:10.3390/healthcare9080973)

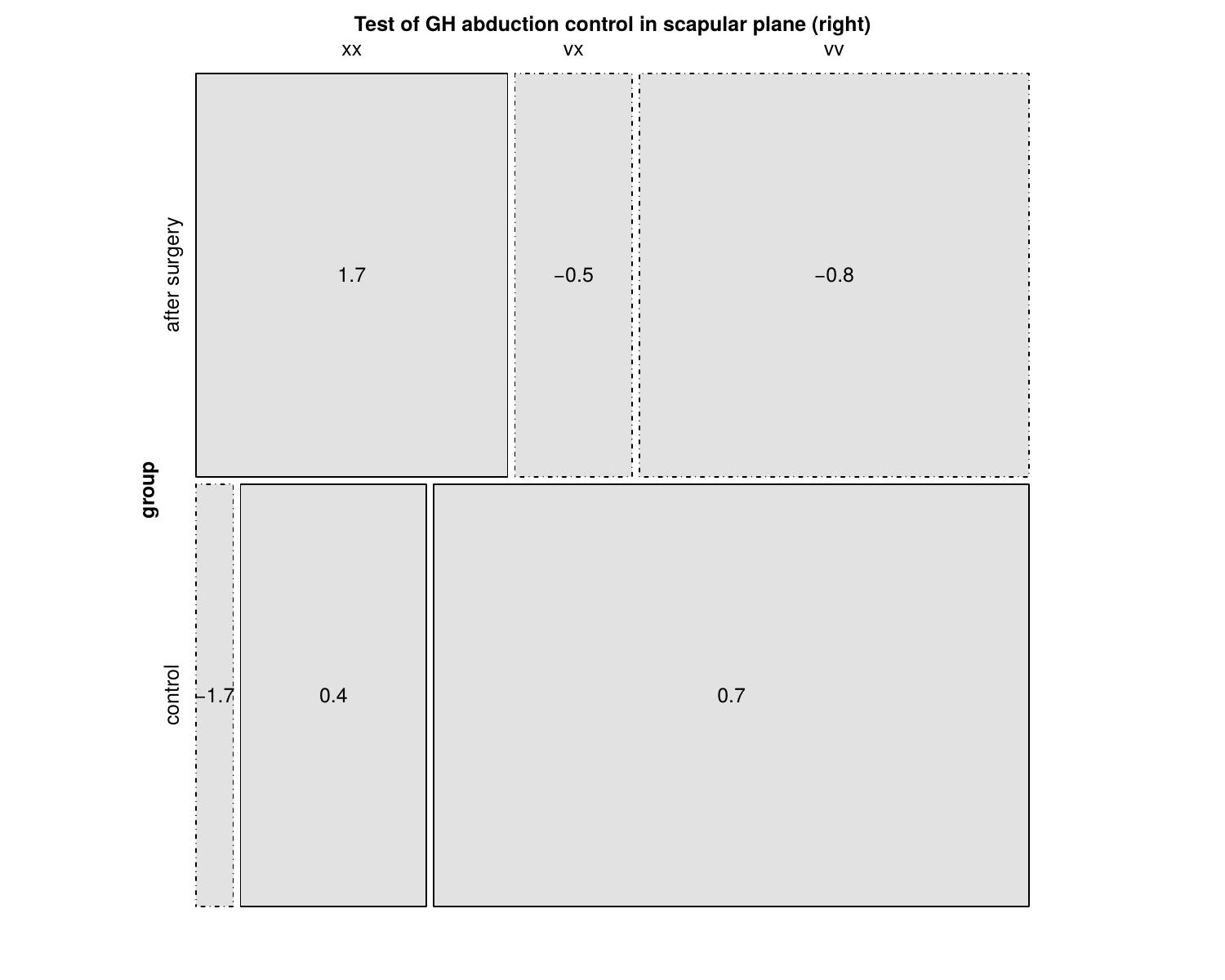

Supplement: Supplementary file 1 [file healthcare-09-00973-s001.zip › Figure S1. Test of GH (glenohumeral) abduction control in scapular plane, right upper extremity..jpg]

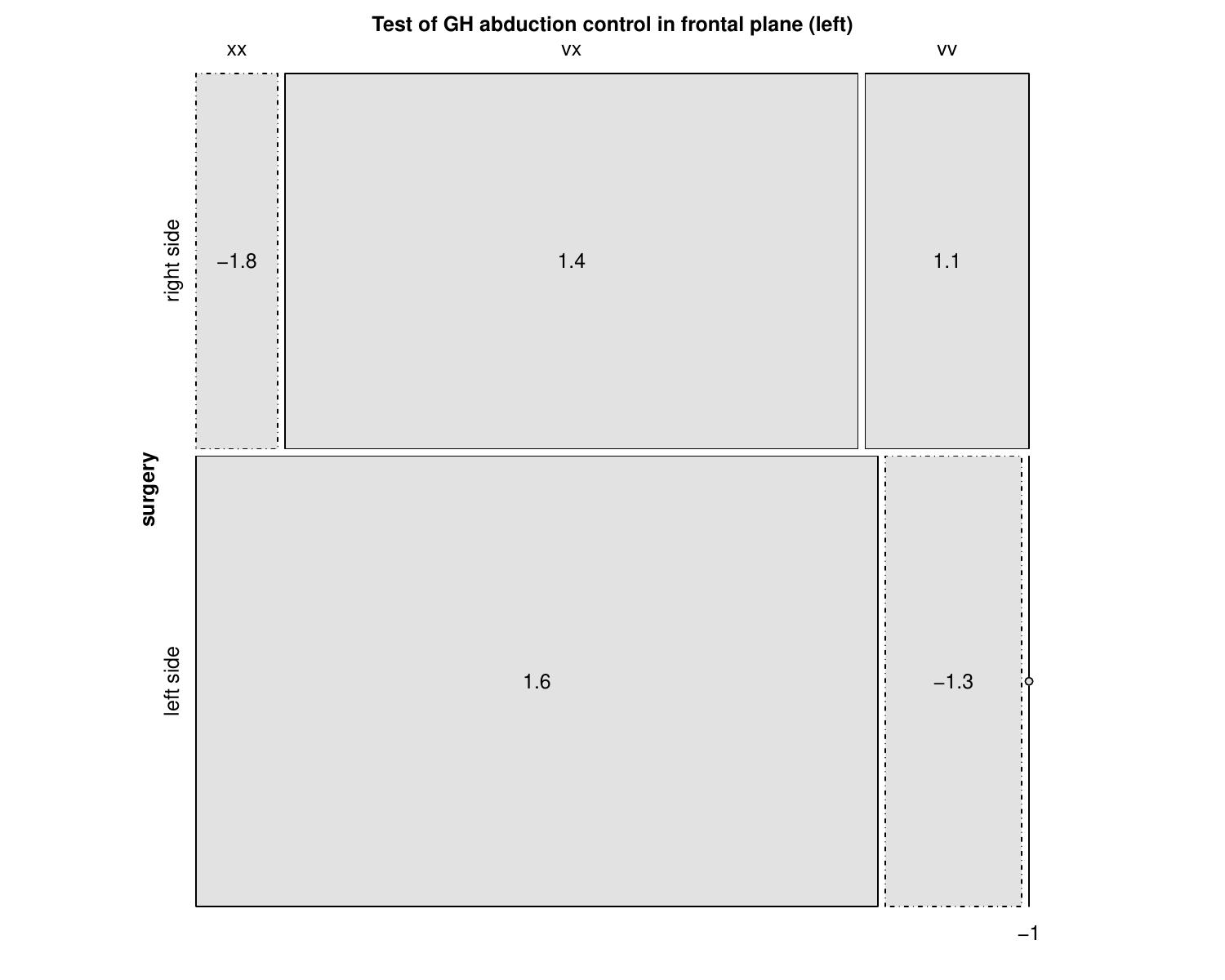

Supplement: Supplementary file 1 [file healthcare-09-00973-s001.zip › Figure S10. Test of GH (glenohumeral) abduction control in frontal plane, left upper extremity..jpg]

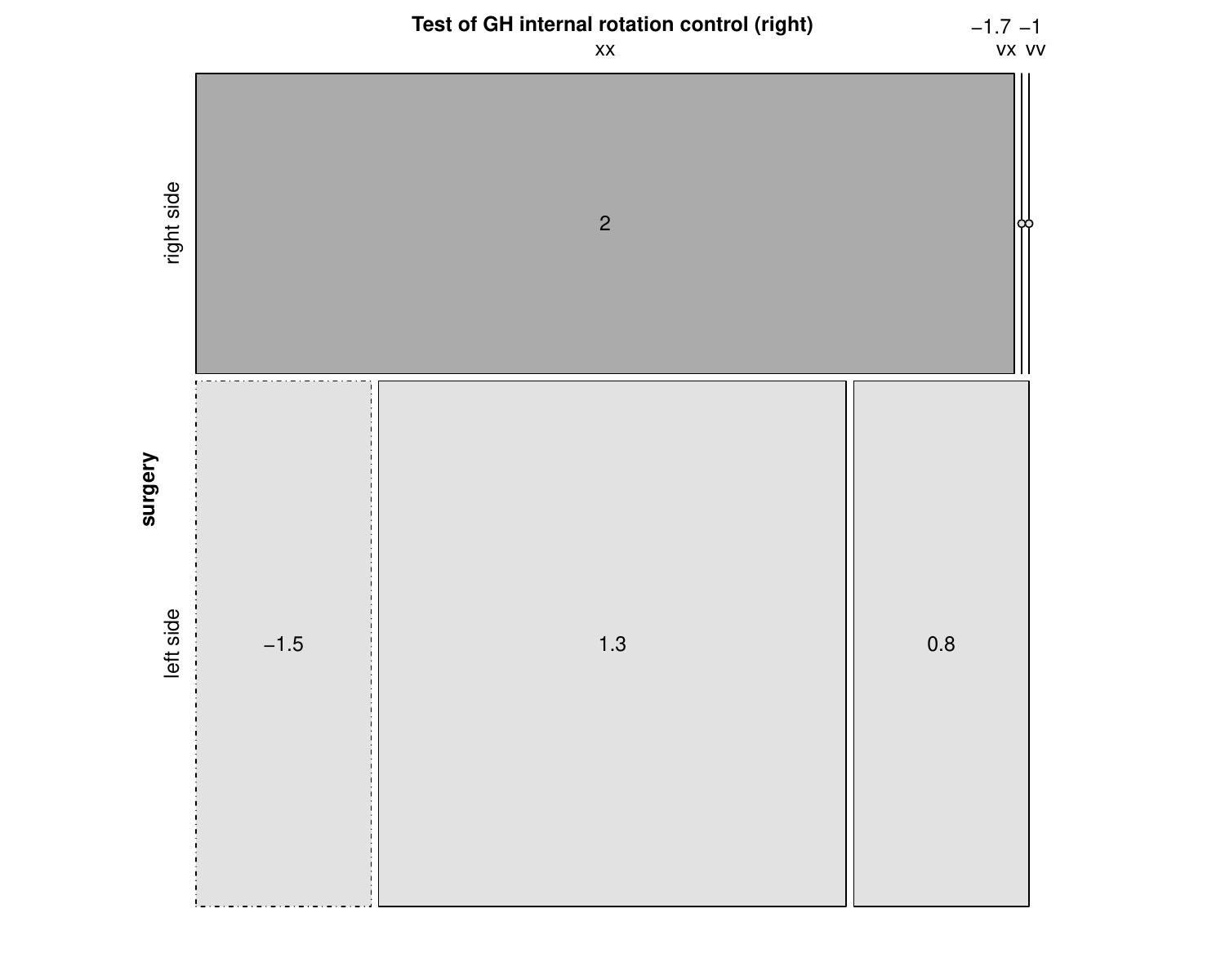

Supplement: Supplementary file 1 [file healthcare-09-00973-s001.zip › Figure S11. Test of GH (glenohumeral) internal rotation control, right upper extremity..jpg]

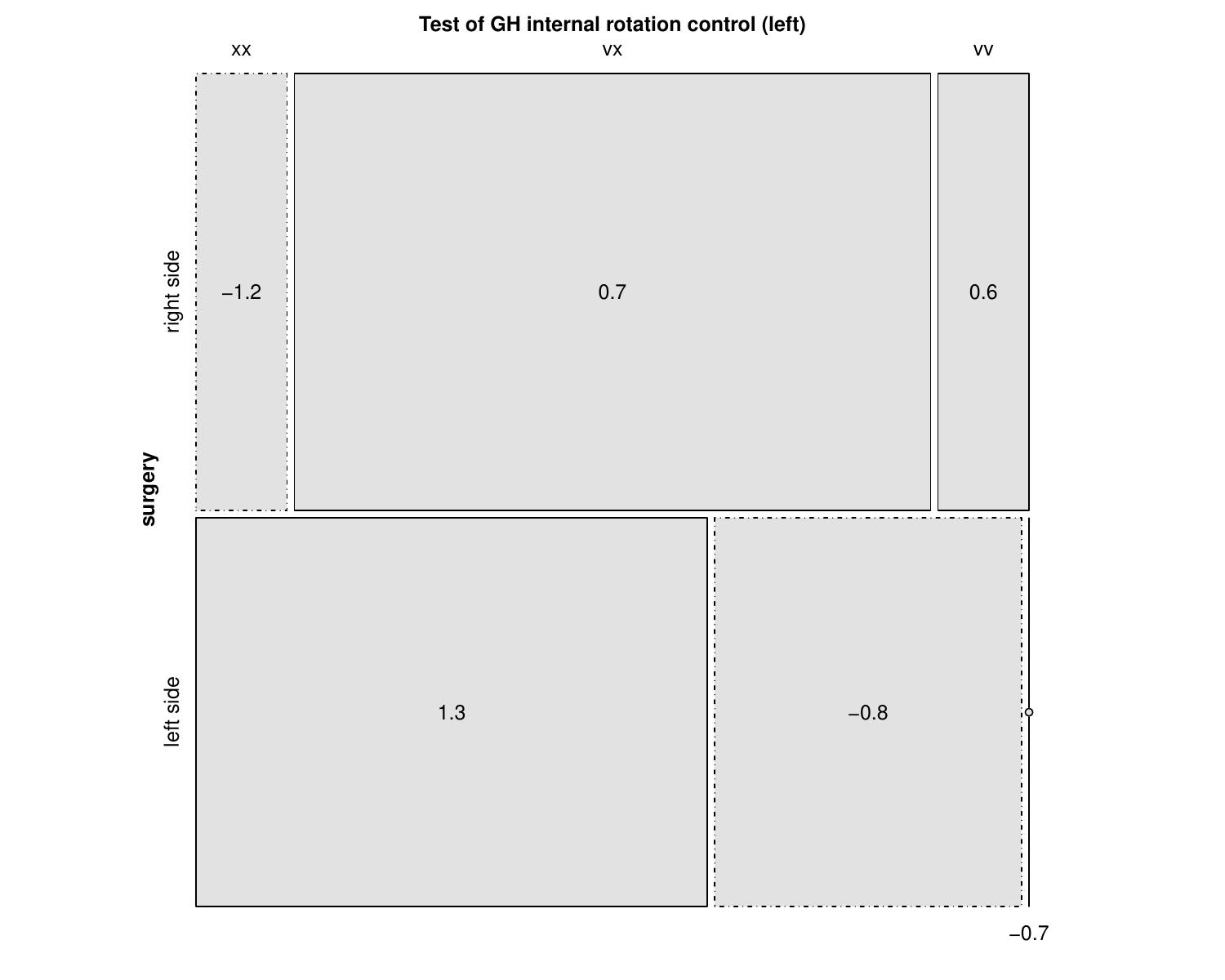

Supplement: Supplementary file 1 [file healthcare-09-00973-s001.zip › Figure S12. Test of GH (glenohumeral) internal rotation control, left upper extremity..jpg]

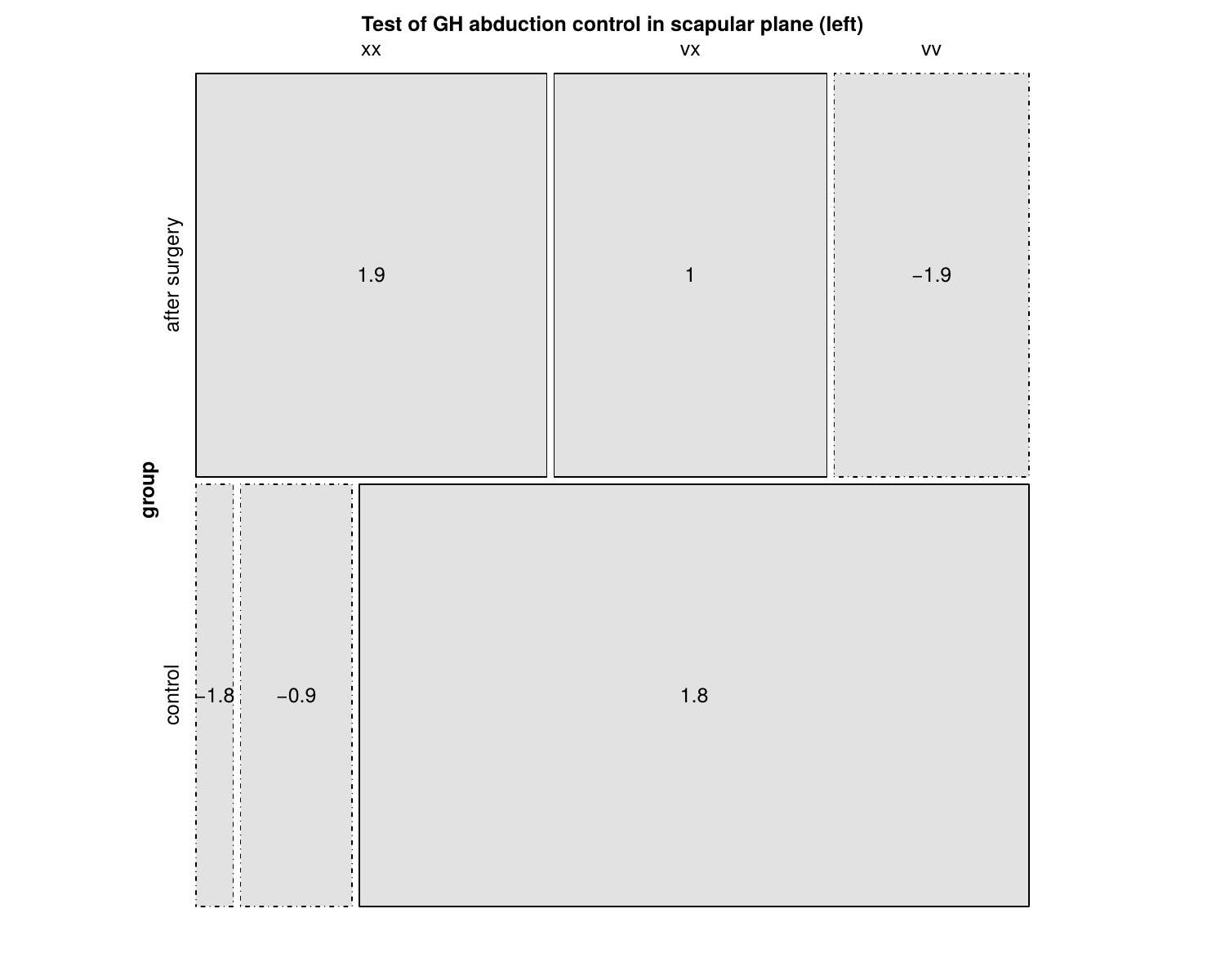

Supplement: Supplementary file 1 [file healthcare-09-00973-s001.zip › Figure S2. Test of GH (glenohumeral) abduction control in scapular plane, left upper extremity..jpg]

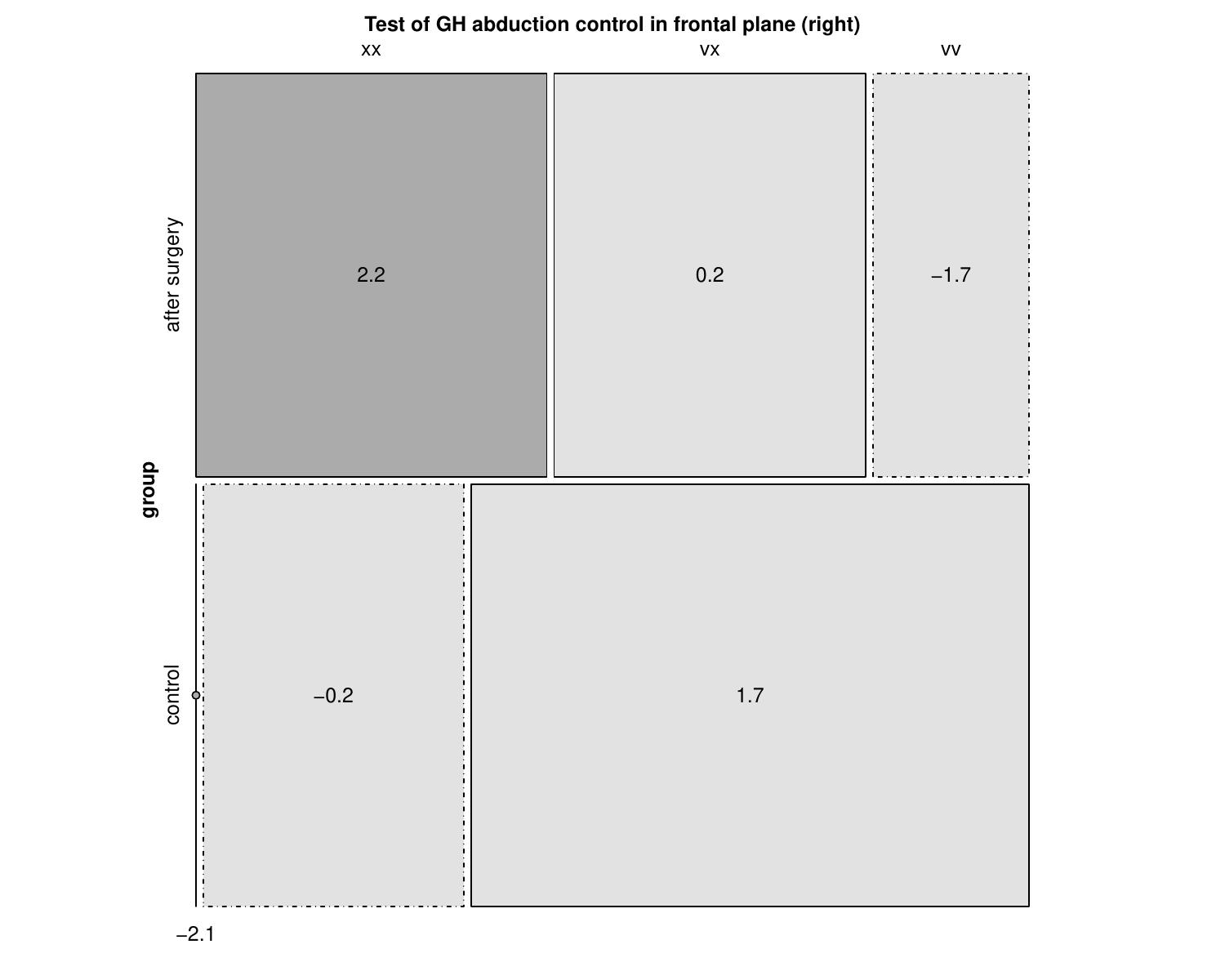

Supplement: Supplementary file 1 [file healthcare-09-00973-s001.zip › Figure s3. Test of GH (glenohumeral) abduction control in frontal plane, right upper extremity.jpg]

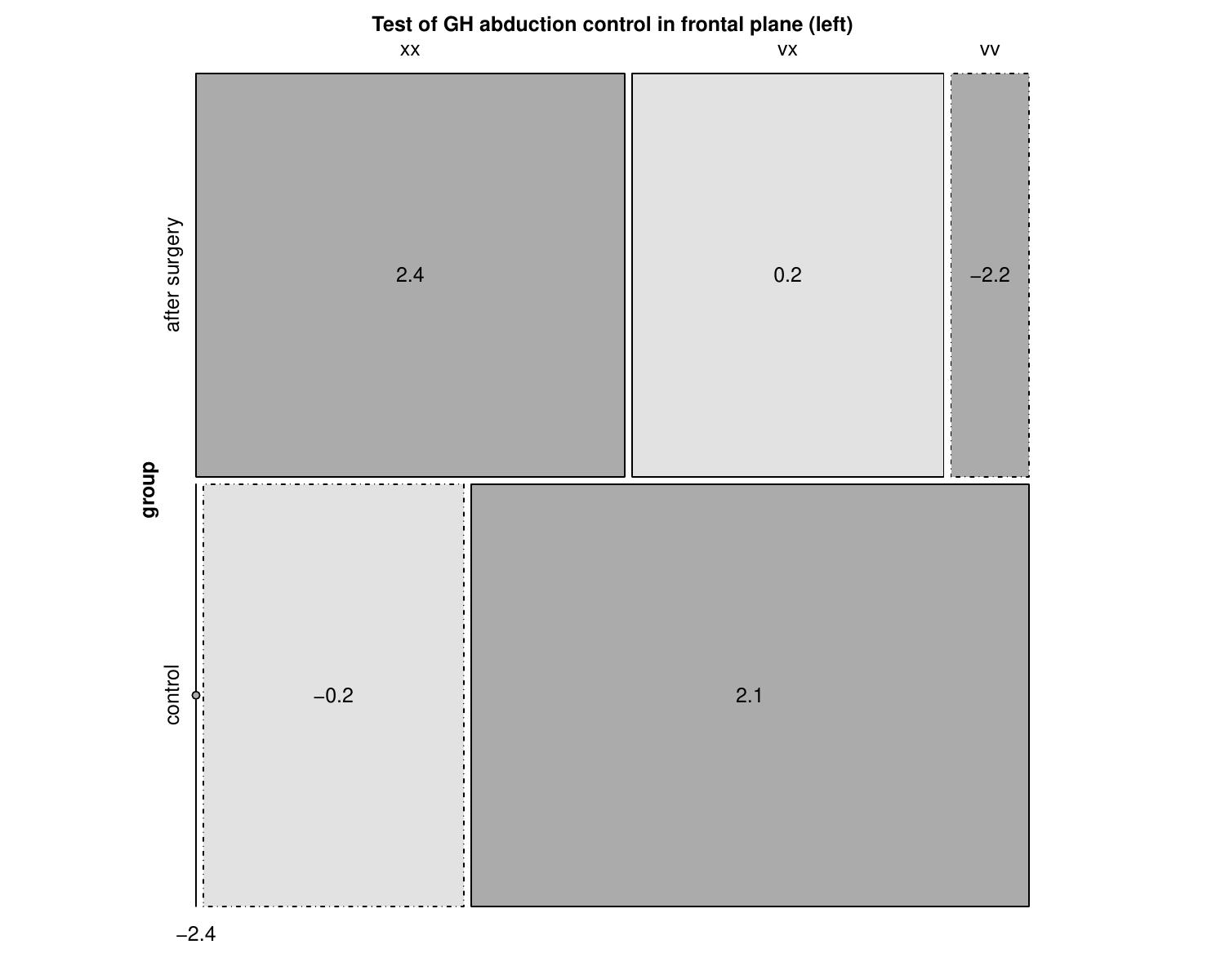

Supplement: Supplementary file 1 [file healthcare-09-00973-s001.zip › Figure S4. Test of GH (glenohumeral) abduction control in frontal plane, left upper extremity..jpg]

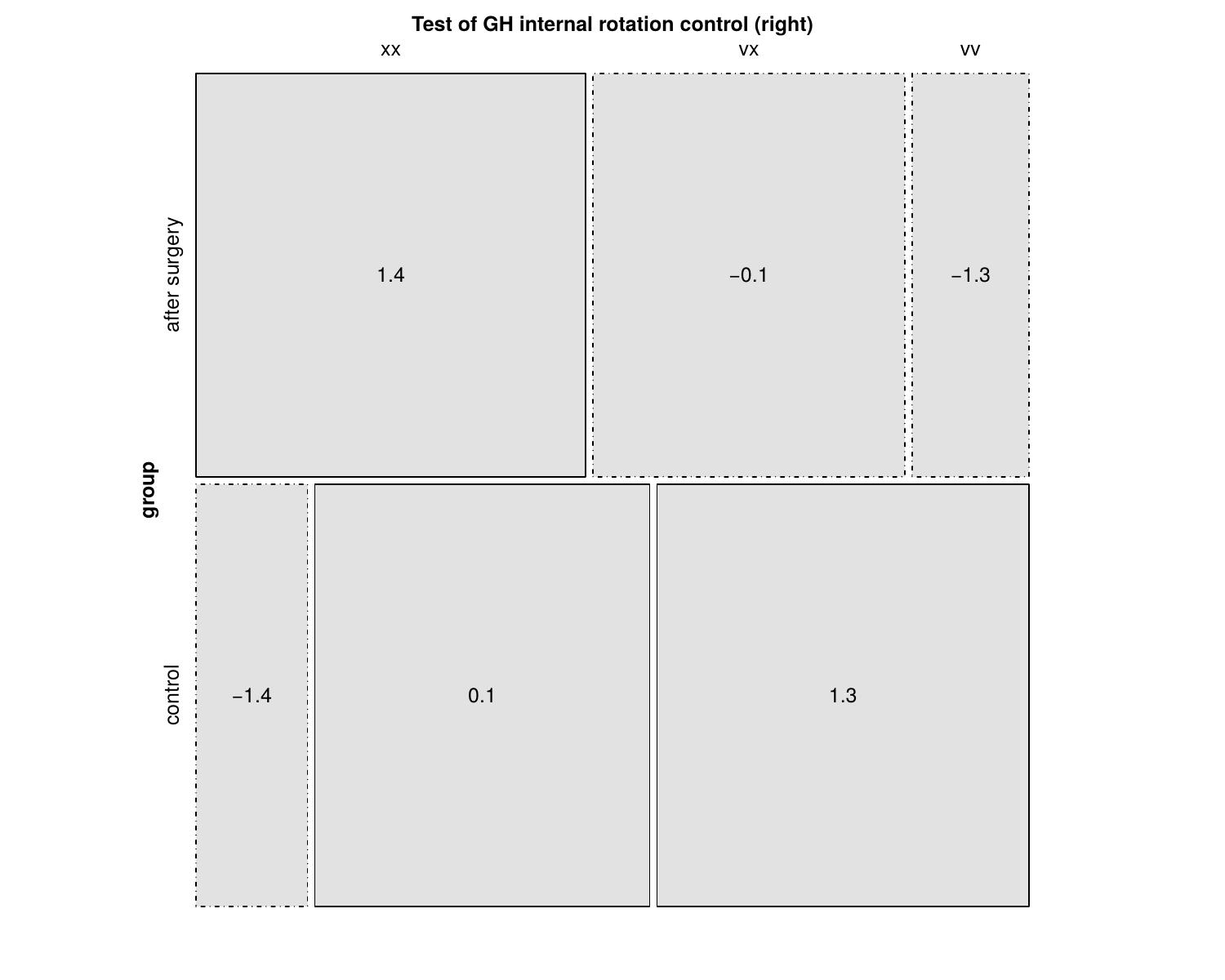

Supplement: Supplementary file 1 [file healthcare-09-00973-s001.zip › Figure S5. Test of GH (glenohumeral) internal rotation control, right upper extremity..jpg]

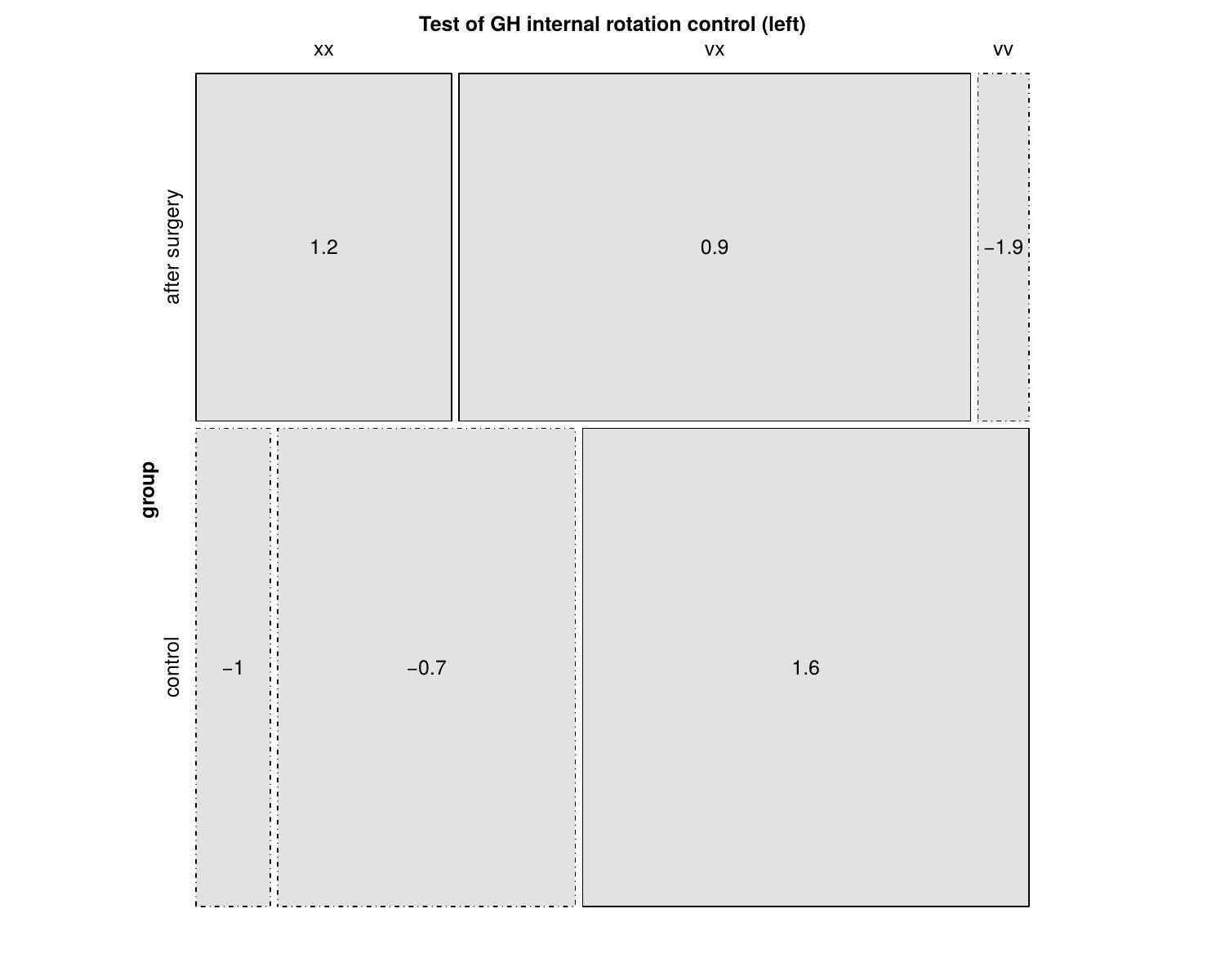

Supplement: Supplementary file 1 [file healthcare-09-00973-s001.zip › Figure S6. Test of GH (glenohumeral) internal rotation control, left upper extremity..jpg]

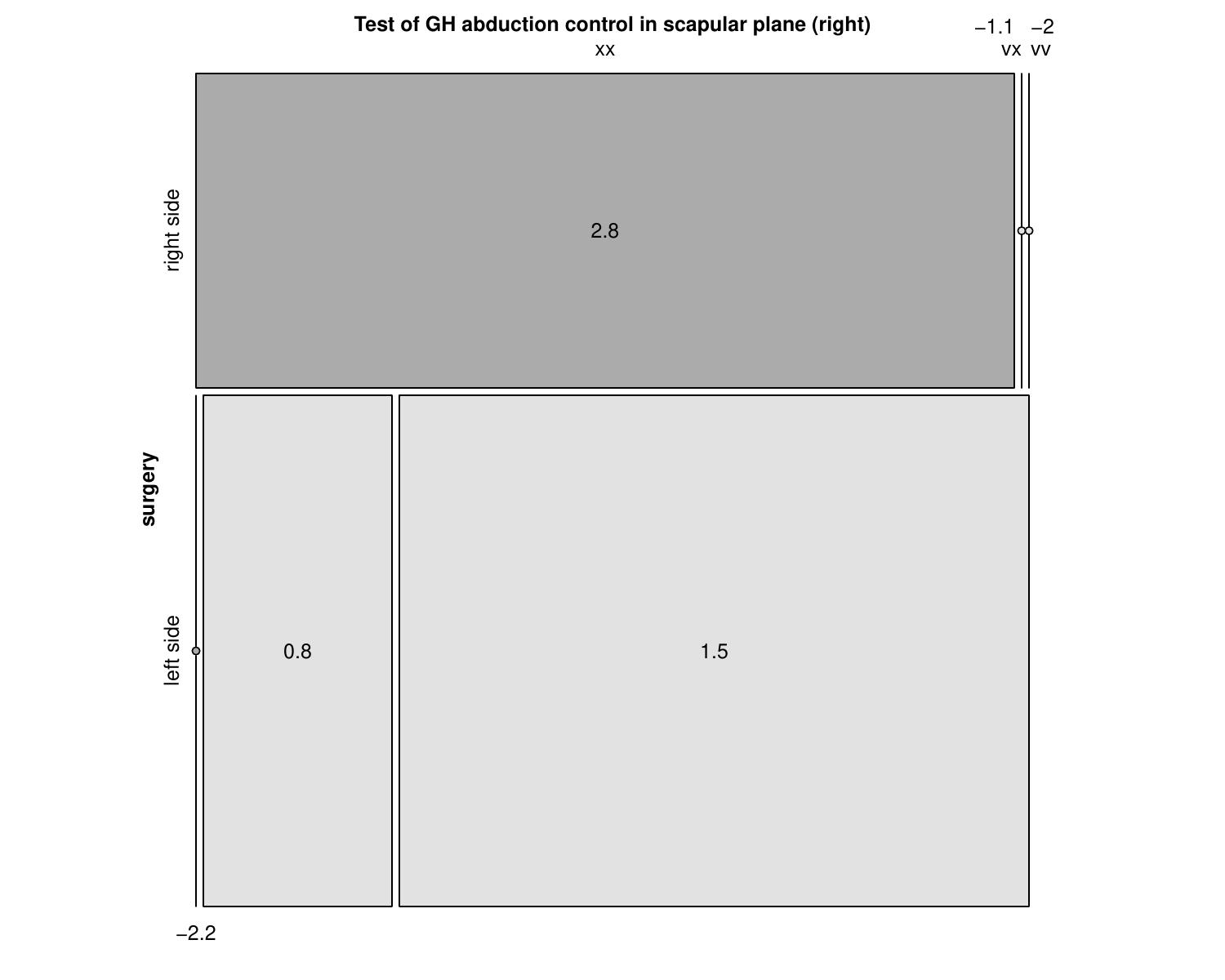

Supplement: Supplementary file 1 [file healthcare-09-00973-s001.zip › Figure S7. Test of GH (glenohumeral) abduction control in scapular plane, right upper extremity..jpg]

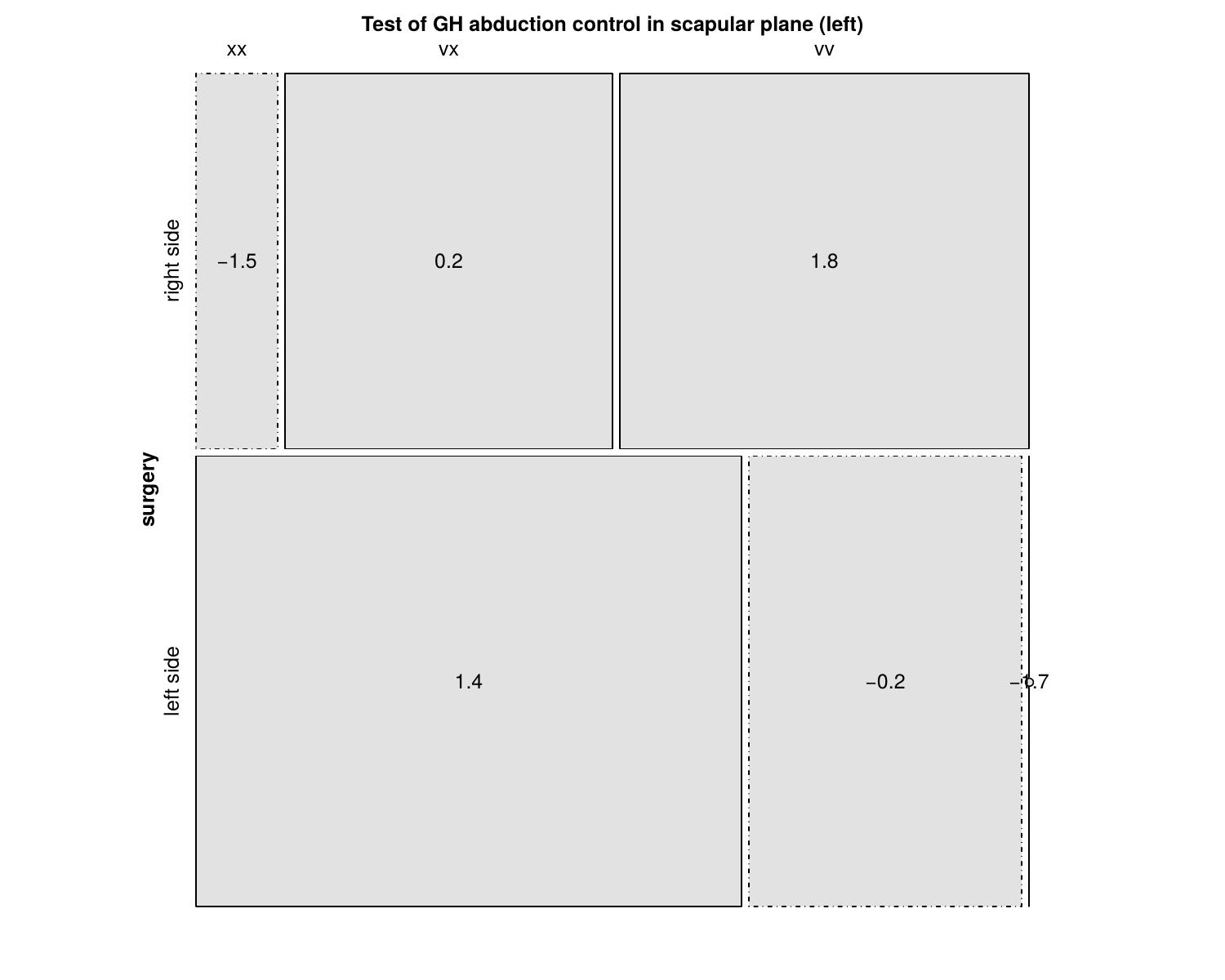

Supplement: Supplementary file 1 [file healthcare-09-00973-s001.zip › Figure S8. Test of GH (glenohumeral) abduction control in scapular plane, left upper extremity..jpg]

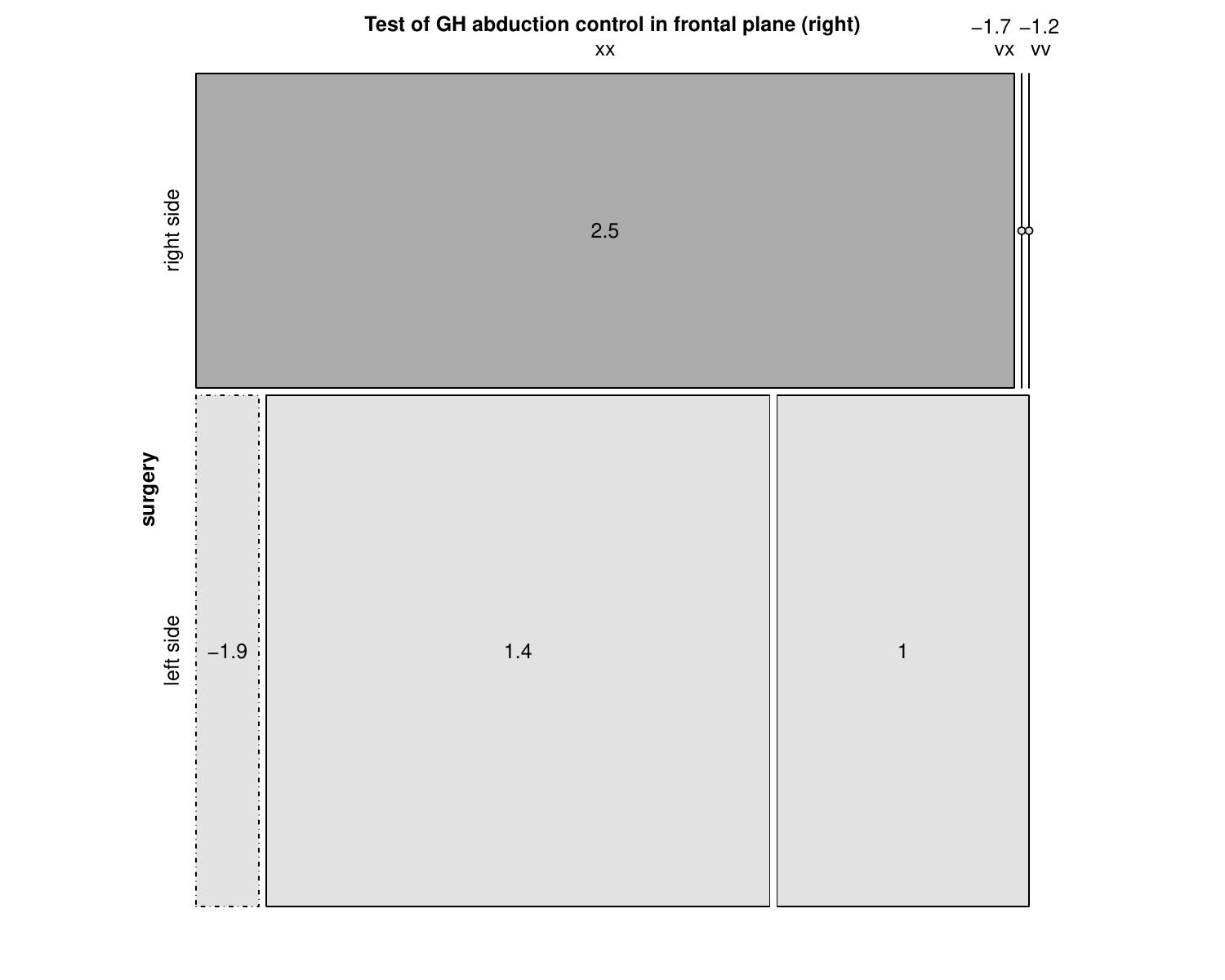

Supplement: Supplementary file 1 [file healthcare-09-00973-s001.zip › Figure S9. Test of GH (glenohumeral) abduction control in frontal plane, right upper extremity..jpg]
